# Supplementary material for: A Food for All Seasons: Stability of Food Preferences in Gorillas across Testing Methods and Seasons
Source: Animals (Basel). 2022 Mar 9;12(6):685. doi: 10.3390/ani12060685 (PMC8944577; doi:10.3390/ani12060685)
Supplement: Supplementary file 1 [file animals-12-00685-s001.zip › animals-1598825-supplementary.pdf]

Table S1: Nutritional Information of Foods

Food	Serving Size	Calories	Calories From Fat	Total Fat		Sodium		Potassium	
				g	dv	mg	dv	mg	dv
Apricots	100g	48		0.39		1		259	
Apples	(242 g/8 oz)	130	0	0	0	0	0	260	7
Asparagus	(93 g/3.3 oz)	20	0	0	0	0	0	230	7
Bananas	(126 g/4.5 oz)	110	0	0	0	0	0	450	13
Beets	100g	43		0.17		78		325	
Broccoli	(148 g/5.3 oz)	45	0	0.5	1	80	3	460	13
Brussel Sprouts	100g	43		0.3		25		389	
Cantaloupe	(134 g/4.8 oz)	50	0	0	0	20	1	240	7
Carrots	(78 g/2.8 oz)	30	0	0	0	60	3	250	7
Cauliflower	(99 g/3.5 oz)	25	0	0	0	30	1	270	8
Celery	(110 g/3.9 oz)	15	0	0	0	115	5	260	7
Cucumbers	(99 g/3.5 oz)	10	0	0	0	0	0	140	4
Tomatoes	(148 g/5.3 oz)	25	0	0	0	20	1	340	10
Eggplant	100g	25		0.18		2		229	
Green Onions	(25 g/0.9 oz)	10	0	0	0	10	0	70	2
Grapefruit	(154 g/5.5 oz)	60	0	0	0	0	0	160	5
Honeydew Melon	(134 g/4.8 oz)	50	0	0	0	30	1	210	6
Green Beans	(83 g/3.0 oz)	20	0	0	0	0	0	200	6
Kiwi	(148 g/5.3 oz)	90	10	1	2	0	0	450	13
Mango	100g	60		0.38		1		168	
Orange	(154 g/5.5 oz)	80	0	0	0	0	0	250	7
Papaya	100g	43		0.26		8		182	
Parsnips	100g	75		0.3		10		375	
Peach	(147 g/5.3 oz)	60	0	0.5	1	0	0	230	7
Pear	(166 g/5.9 oz)	100	0	0	0	0	0	190	5
Pineapple	(112 g/4 oz)	50	0	0	0	10	0	120	3
Plum	(151 g/5.4 oz)	70	0	0	0	0	0	230	7
Potato	(148 g/5.3 oz)	110	0	0	0	0	0	620	18
Radish	(85 g/3.0 oz)	10	0	0	0	55	2	190	5
Red Onions	(148 g/5.3 oz)	45	0	0	0	5	0	190	5
Romaine Lettuce	(85 g/3.0 oz)	15	0	0	0	35	1	170	5

Rutabaga	100g	37		0.16		12		305	
Strawberries	(147 g/5.3 oz)	50	0	0	0	0	0	170	5
Sweet Peppers	(148 g/5.3 oz)	25	0	0	0	40	2	220	6
Turnips	100g	28		0.1		67		191	
Watermelon	(280 g/10.0 oz)	80	0	0	0	0	0	270	8
Grapes	(126 g/4.5 oz)	90	0	0	0	15	1	240	7
Yam	(130 g/4.6 oz)	100	0	0	0	70	3	440	13

Information gathered from fda.gov and https://fdc.nal.usda.gov/fdc-app.html#/food-search (shaded)

Total Carbs		Dietary Fiber		Sugars	Protein	Vit A	Vit C	Calcium	Iron
g	dv	g	dv	g	g	dv	dv	dv	dv
11.1		2		9.24	1.4				
34	11	5	20	25	1	2	8	2	2
4	1	2	8	2	2	10	15	2	2
30	10	3	12	19	1	2	15	0	2
9.56		2.8		6.76	1.61				
8	3	3	12	2	4	6	220	6	6
8.95		3.8		2.2	3.38				
12	4	1	4	11	1	120	80	2	2
7	2	2	8	5	1	110	10	2	2
5	2	2	8	2	2	0	100	2	2
4	1	2	8	2	0	10	15	4	2
2	1	1	4	1	1	4	10	2	2
5	2	1	4	3	1	20	40	2	4
5.88		3		3.53	0.98				
2	1	1	4	1	0	2	8	2	2
15	5	2	8	11	1	35	100	4	0
12	4	1	4	11	1	2	45	2	2
5	2	3	12	2	1	4	10	4	2
20	7	4	16	13	1	2	240	4	2
15		1.6		13.7	0.82				
19	6	3	12	14	1	2	130	6	0
10.8		1.7		7.82	0.47				
18		4.9		4.8	1.2				
15	5	2	8	13	1	6	15	0	2
26	9	6	24	16	1	0	10	2	0
13	4	1	4	10	1	2	50	2	2
19	6	2	8	16	1	8	10	0	2
26	9	2	8	1	3	0	45	2	6
3	1	1	4	2	0	0	30	2	2
11	4	3	12	9	1	0	20	4	4
2	1	1	4	1	1	130	6	2	4

8.62		2.3		4.46	1.08				
11	4	2	8	8	1	0	160	2	2
6	2	2	8	4	1	4	190	2	4
6.43		1.8		3.8	0.9				
21	7	1	4	20	1	30	25	2	4
23	8	1	4	20	0	0	2	2	0
23	8	4	16	7	2	120	30	4	4
